# First Report of *Colletotrichum fructicola* Causing Fruit Rot and Leaf-Tip Dieback on Pineapple in Northern Thailand

**DOI:** 10.3390/plants12040971

**Published:** 2023-02-20

**Authors:** Alireza Armand, Kevin David Hyde, Ruvishika Shehali Jayawardena

**Affiliations:** 1 Center of Excellence in Fungal Research, Mae Fah Luang University, Chiang Rai 57100, Thailand; 2 School of Science, Mae Fah Luang University, Chiang Rai 57100, Thailand; 3 Innovative Institute for Plant Health, Zhongkai University of Agriculture and Engineering, Guangzhou 510225, China

**Keywords:** *Ananas comosus*, phylogeny, plant pathogen, new record, taxonomy, tropical fruit

## Abstract

Pineapple is one of the most economically important fruits in tropical countries, particularly in Thailand. Canned pineapple is currently Thailand’s main exported commodity to many countries, including the United States, Russia, Germany, Poland, and Japan. Fungal diseases are considered a permanent threat to fruits in the pre- and post-harvest stages, leading to considerable economic losses. Fungal disease is one of the primary causes of massive yield losses in pineapples around the world. *Colletotrichum* species are the most common fungal pathogens affecting different tropical fruits. Although there are many reports regarding *Colletotrichum* species associated with pineapple, they do not have molecular data to confirm species identification. However, the occurrence of *Colletotrichum* species on pineapple has not been reported in Thailand so far. In this study, we isolated and identified *Colletotrichum fructicola* on pineapple in northern Thailand and have proven its pathogenicity to the host. This is the first report of the occurrence of *Colletotrichum* in pineapple, based on morpho-molecular approaches.

## 1. Introduction

Pineapple (*Ananas comosus* (L.) Merr.) is one of the edible and nutritious fruits of the Bromeliaceae, grown in tropical and subtropical countries [1]. Asia, South Central America, and Africa are the world’s leading areas producing this fruit [2]. Pineapple is the second largest tropical crop in the world [2] and the third most consumed fruit worldwide [3]. Brazil, China, Costa Rica, India, the Philippines, and Thailand are the top pineapple-producing countries [1]. Pineapple could be eaten as a fresh fruit or selected as a basic raw ingredient used in the confectionery industry [4,5,6]. The fruit contains immense nutrients and is abundant in vitamins A, C, B1, and B6 [7,8,9]. It also has proteins, carbohydrates, fiber, copper, manganese, and several minerals [5,10].

In Thailand, pineapple products have been regarded as economic commodities for export [11]. Thailand is currently the second-largest supplier of processed pineapple fruit in the world. Production areas for pineapple in Thailand have been divided into northern, northeastern, central, and southern parts [12]. Most cultivated areas are in Prachuap Khiri Khan, Rayong, Ratchaburi, and Chonburi provinces [11]. Pineapple is classified into five groups based on the morphology of the leaf and fruit, namely Abacaxi, Cayenne, Maipure or Perolera, Queen, and Spanish [13]. Among these, Cayenne, Queen, and Spanish are cultivated in Thailand [14]. Nang Lae district in Chiang Rai province is the most pineapple-cultivating area, and Nang Lae (Cayenne group) and Phu Lae (Queen group) are the most common varieties in northern Thailand (http://www.doa.go.th/; https://www.saio.co.th/; accessed on 12 December 2022). The pineapple-growing area in Thailand is around 72,656 hectares, with an annual production of 1,680,884 metric tons. The total canned pineapples exported from Thailand were 290,524 metric tons in 2020, valued at about 345 million US dollars [15].

Diseases are the key elements of significant yield losses in pineapples across the world [1]. Among fungal pathogens, *Colletotrichum* species are the most important fungi responsible for the diseases of tropical and sub-tropical fruits [16]. Many *Colletotrichum* species have been reported from different hosts in Thailand such as *C. aenigma* [17], *C. aeschynomenes* [18], *C. artocarpicola* [19], *C. asianum* [20,21,22,23], *C. boninense* [18,24], *C. brevisporum* [16,25,26,27], *C. chiangraiense* [24,28], *C. cordylinicola* [21,29,30], *C. endophytica* [31,32,33,34], *C. orchidearum* [24,25], *C. orchidophilum* [24], *C. plurivorum* [35], and *C. siamense* [16,21,24,36,37], majority belonging to gloeosporioides species complex. *Colletotrichum fructicola* is one of the most invasive species and has been reported as the causal agent of anthracnose, leaf spots and bitter rots in more than 90 plant species [38]. It was originally isolated from *Coffea arabica* in northern Thailand [39]. It has also been reported from *Capsicum annuum* [24], *Carica papaya* [20,24], *Cymbopogon citratus* [16,24], *Dendrobium* sp. [24], *Dimocarpus longan* [20,24,29], *Freycinetia* sp. [24], *Pandanus* sp. [24], *Pennisetum purpureum* [16,24] in Thailand. Mealybug wilt-associated virus, bacterial heart rot, fruit collapse, butt rot, fruitlet core rot, black rot, yeasty, and fusariosis are the main diseases of pineapple, discussed by Sapak et al., (2021). Despite the importance of pineapple in Thailand, studies for the isolation and identification of fungal pathogens associated with pineapple have not been conducted in Thailand.

## 2. Results

### 2.1. Morphological Studies

Following 7–14 days of incubation, morphological features including culture (color and growth rate) and microscopic features (conidiogenous cells and conidial measurements, appressoria measurements) were recorded for P76 (MFLU 22-0302) and P76-3 (MFLU 22-0303). The two strains, P76 and P76-3 were isolated from rotting pineapple fruit and leaf dieback, respectively. Morphological comparisons of P76 and P76-3 were performed on 14-day-old cultures grown on PDA at 25 °C ± 2 °C. There were minor differences in size of conidia and conidiogenous cells and also in appressoria shape which are very common in a specific species within the *C. gloeosporioides* species complex. Finally, these two isolates were identified as *C. fructicola* based on morpho-molecular evidence.

### 2.2. Phylogenetic Analyses

**Figure 1:**
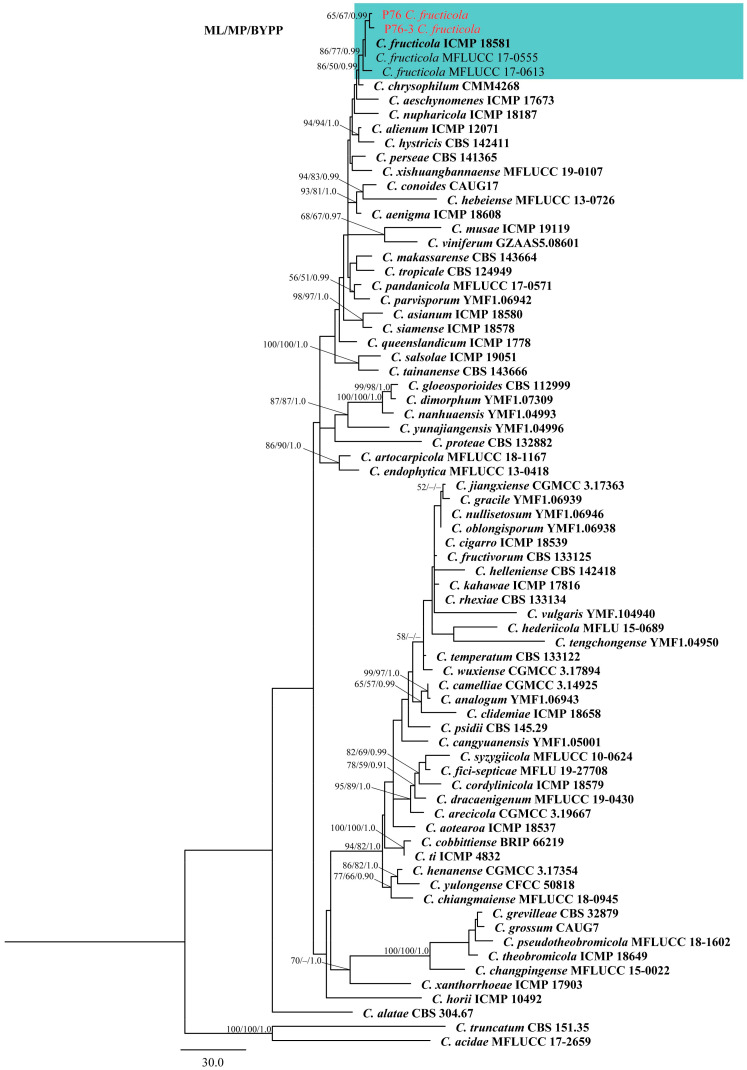
Maximum parsimony tree of the *Colletotrichum gloeosporioides* species complex generated by analysis of combined ITS, *ACT*, *CHS-1*, *GAPDH*, and *TUB2* sequence data. The tree was rooted with *Colletotrichum truncatum* (CBS 151.35) and *Colletotrichum acidae* (MFLUCC 17.2659). Maximum likelihood and maximum parsimony bootstrap values ≥ 50% and bayesian posterior probabilities ≥ 0.90 are shown near the nodes, respectively. Type strains are in bold and the newly generated isolates are in red.

The five-locus (ITS, *ACT*, *GAPDH*, *CHS-1*, and *TUB2*) phylogenetic analysis included 73 reference isolates [40,41,42]. The phylogenetic tree consisted of 71 ingroup and 2 outgroup taxa (*Colletotrichum truncatum*, CBS 151.35 and *C. acidae*, MFLUCC 17-2659). The data matrix contained a total of 1670 characters, of which 269 were parsimony-uninformative and 384 were parsimony-informative. The most parsimonious tree (Tree Length (TL) = 1321, Consistency Index (CI) = 0.673, Retention Index (RI) = 0.824, Rescaled Consistency Index (RC) = 0.554, Homoplasy Index (HI) = 0.327) was presented (Figure 1). The ML, MP and BYPP trees were identical in topology. The best-scoring RAxML tree with final optimization showed a likelihood value of −9834.854352. The dataset comprised 754 distinct alignment patterns, with 5.31% of characters being gaps or undetermined. Estimated base frequencies were as follows: A = 0.229261, C = 0.299900, G = 0.241493, T = 0.229347, with substitution rates AC = 1.133302, AG = 2.907494, AT = 1.290422, CG = 0.902726, CT = 4.957643, GT = 1.000000. The gamma distribution shape parameter is 0.422238 and the tree length is 0.963648. Based on the phylogenetic analysis, strains P76 and P76-3 clustered with *C. fructicola*, showing 86/77/0.99 ML, MP, and BYPP values, respectively (Figure 1). The base pair differences between these two strains and the ex-type of *C. fructicola* (ICMP 18581) were shown (Table 1).

### 2.3. Taxonomy

**Figure 2:**
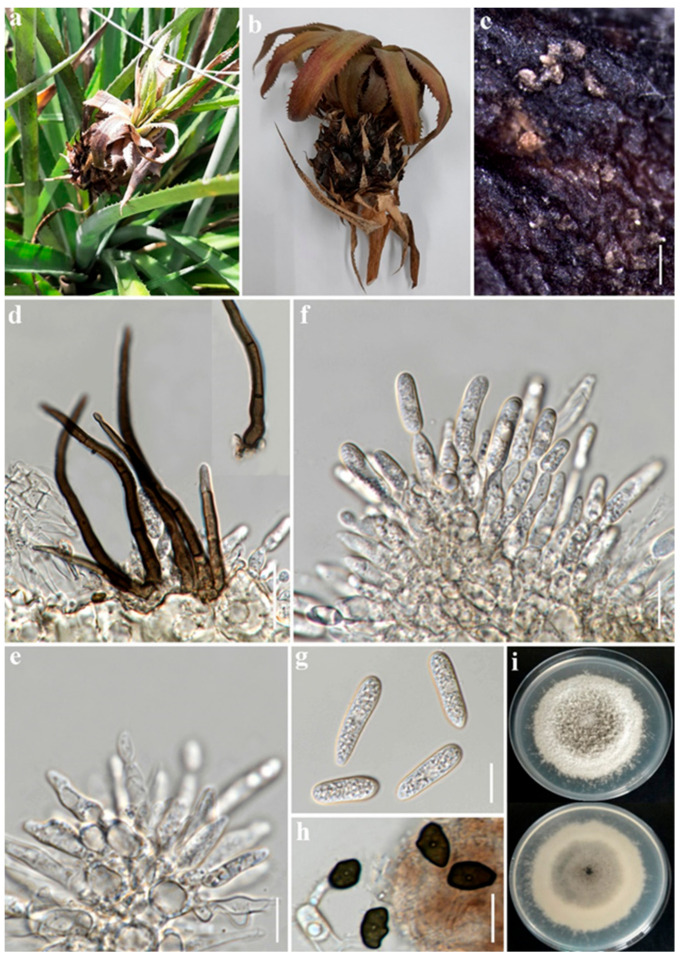
*Colletotrichum fructicola* P76 (MFLU 22-0302); (**a**,**b**). Rotted pineapple fruit; (**c**). Acervuli on the fruit; (**d**). Setae; (**e**). Conidiophores; (**f**). Conidiogenous cells and conidial attachment; (**g**). Conidia; (**h**). Appressoria; (**i**). Upper and reverse view of colony on PDA. Scale bars: (**c**) = 200 µm, (**d**) = 20 µm, (**e**–**h**) = 10 µm.

**Figure 3:**
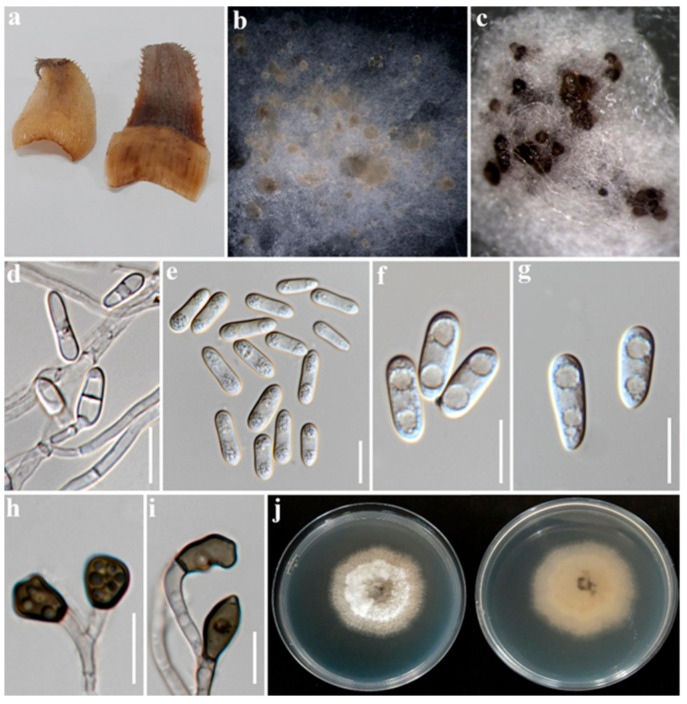
*Colletotrichum fructicola* P76-3 (MFLUCC 22-0182); (**a**). Symptomatic leaves; (**b**). Conidial masses on PDA; (**c**). Acervuli on PDA; (**d**). Conidial attachment; (**e**–**g**). Conidia; (**h**,**i**). Appressoria; (**j**). Upper and reverse view of colony on PDA. Scale bars: (**d**–**i**) 10 µm.

***Colletotrichum fructicola*** Prihast., L. Cai & K.D. Hyde (2009) (Figure 2 and Figure 3).

Index fungorum number: IF 515409; Faces of Fungi number: FoF 06767.

Associated with pineapple fruit rot and leaf dieback. Sexual morph: Not observed. Asexual morph: *Vegetative hyphae* hyaline, smooth-walled, septate, branched. *Conidiomata acervular*, dark brown, bearing conidial mass, and setae. *Setae* brown to dark brown, smooth-walled, 2–4 septate, 38–83 μm long ($\bar{x}$ = 59.5 μm, *n* = 6), base cylindrical, 3–5 μm diam. ($\bar{x}$ = 4.5 µm, *n* = 6), tip acute or obtuse. *Conidiophores* rarely observed, hyaline, septate, branched, cylindrical to inflated. *Conidiogenous cells* hyaline, cylindrical or clavate, 12–25 × 3–4.5 μm ($\bar{x}$ = 18 × 3.5 µm, *n* = 20). *Conidia* hyaline, aseptate, smooth-walled, cylindrical, rounded at apex, sub-acute at base, guttulate, 12.5–19 × 4.5–6 μm ($\bar{x}$ = 16 × 5 µm, *n* = 30).

Culture characteristics: Colonies on PDA 65–85 mm in diam. after 7 days at 28 °C, velvety, circular, undulate; surface pale grey in center and white in margin, becoming grey with age; reverse same color. Colonies on OA 60–71 mm in diam. after 7 days, cottony, slightly raised, entire; surface white to whitish grey; reverse same color. *Appressoria* produced on slide culture, brown to dark brown, irregular in shape, undulate, 7–9 × 4.5–6 μm ($\bar{x}$ = 7.8 × 5 µm, *n* = 15), producing on hyphae and conidia.

Material examined: Thailand, Chiang Rai Province, Mueang Chiang Rai District, Ban Du Sub-district. On pineapple rotting fruit, 27 June 2022, Alireza Armand, P76 (MFLU 22-0302), living culture, MFLUCC 22-0181. On pineapple leaf dieback, 27 June 2022, Alireza Armand, P76-3 (MFLU 22-0303), living culture, MFLUCC 22-0182.

Notes: The species within the gloeosporioides species complex are mainly distinguished by producing cylindrical conidia with rounded ends, tapering slightly towards the base [16]. The strain P76 was isolated directly from rotting pineapple, whereas P76-3 was obtained by tissue isolation from a fresh leaf with tip dieback symptoms. Based on the phylogenetic tree (Figure 1), isolates P76 (MFLUCC 22-0182) and P76-3 (MFLU 22-0303) clustered with *C. fructicola* strains with 86/77/0.99 ML, MP, and BYPP values, respectively. Morphologically, P76 and P76-3 are similar. However, P76 produced slightly larger conidia than P76-3 (13–19 × 4.5–6 μm in P76 vs. 12.5–17.5 × 4–6 μm in P76-3). The conidial shape was slightly different, as P76 produced conidia with obtuse ends, whereas P76-3 mostly produced conidia with rounded ends. However, morphological comparison with the ex-type of *C. fructicola* revealed no significant differences between the type strain and our isolates (P76, P76-3) [39].

### 2.4. Pathogenicity Assay

**Figure 4:**
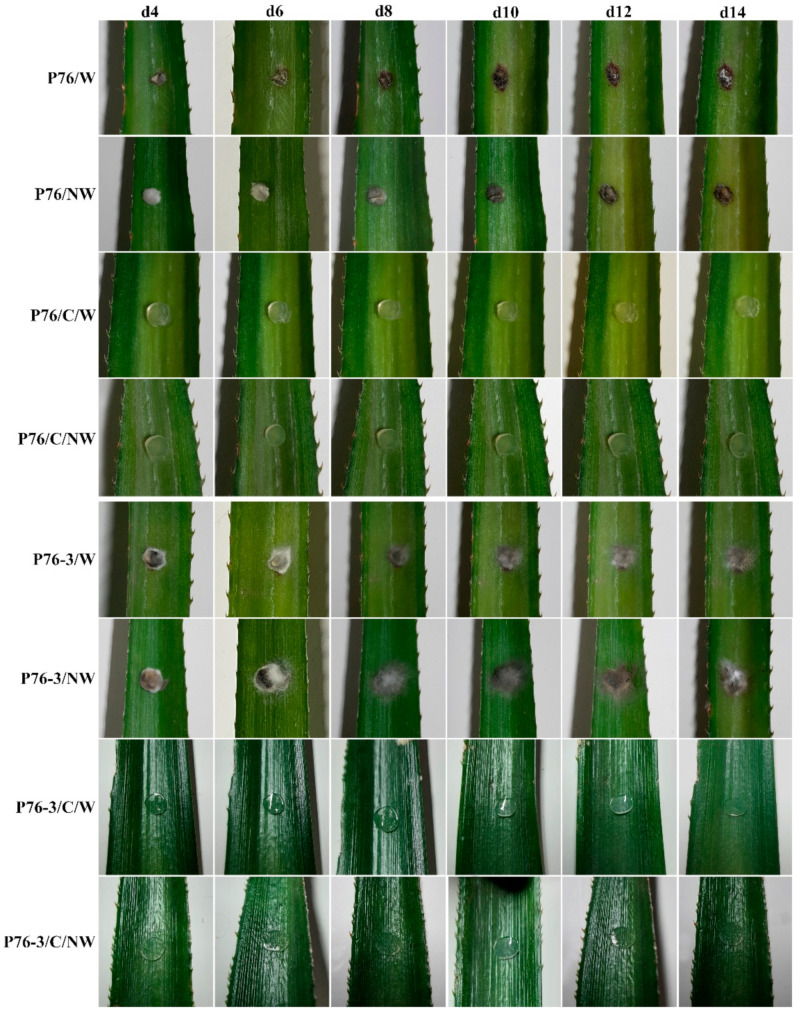
Pathogenicity testing on pineapple leaves. Symptoms (dieback) on days 4 (**d4**), 6 (**d6**), 8 (**d8**), 10 (**d10**), 12 (**d12**), and 14 (**d14**) after inoculation are shown. **C**: Control, **W**: Wounded, **NW**: Non-wounded.

Pathogenicity test results related to strain P76 showed that this strain can cause disease on both wounded and non-wounded host leaves. The wounded leaves inoculated with P76 showed dieback symptoms 4 days after the inoculation, whereas those of the non-wounded leaves showed symptoms 6 days after the inoculation. However, the symptoms continued to spread in both wounded and non-wounded leaves after 10 days. After 11 days, aerial mycelia started to grow on the surface of the symptomatic area in both wounded and non-wounded leaves. (Figure 4, P76/W, P76/NW).

Six days after the inoculation, symptoms on the injured leaves treated with P76-3 were only present at the border of the mycelial plugs (Figure 4, P76-3/W). The non-wounded leaves inoculated with P76-3 remained asymptomatic during the test (Figure 4, P76-3/NW) along with the negative control treatments. The re-isolated fungi were identified as *C. fructicola* according to morphological characteristics.

## 3. Discussion

In this study, the diseased leaves showing dieback symptoms and rotting fruits of pineapple were collected in Chiang Rai province, northern Thailand. Based on a direct isolation from the fruiting bodies (Figure 2) and an indirect isolation of infected leaves via tissue culture (Figure 3), we obtained two *Colletotrichum* isolates. Jayawardena et al., (2021) recommended using a polyphasic approach to identify *Colletotrichum* species [43]. We used both morphological examination and multi-loci molecular analysis for species-level identification. The phylogenetic analysis of a combined dataset of ITS, *ACT*, *CHS-1*, *GAPDH*, and *TUB2* showed that the two isolates of *Colletotrichum* associated with pineapple belong to *C. fructicola* (Figure 1). Morphological studies also confirmed the phylogenetic results. *Colletotrichum* species have a wide host range and geographical distribution worldwide [16,44]. However, the occurrence of *Colletotrichum* species on pineapple has not so far been reported in Thailand. In the USDA host fungal database, there are 12 records of *Colletotrichum* species on *Ananas* sp. [45]. *Colletotrichum ananas* was reported in India [46], while *C. truncatum* was reported to cause leaf-tip dieback in Malaysia [45]. *Colletotrichum gloeosporioides* has been recorded to cause anthracnose on pineapple in China and the United States [45]. *Colletotrichum gloeosporioides* was also recorded from *Ananas* spp. in Brazil on pineapple leaves. *Colletotrichum* sp. was identified in Cuba, India, Korea, Panama, and the West Indies [47]. However, these species were identified based only on morphology.

In recent decades, identification based on morphology has led to the misidentification of fungal pathogens [43]. In plant pathology, correct identification of fungal species is a fundamental step that links information concerning biology, host range, distribution, pathogenicity and food security [43,48], indicating the importance of species identification to barricade future afflictions provoked by these pathogens [49]. Moreover, emerging pathogens have also been increasing threats during the last decade [24]. *Colletotrichum*, being a complex genus, shares overlapping morphological characteristics among species. *Colletotrichum fructicola* belongs to the gloeosporioides species complex, which comprises fruit rots and post-harvest pathogens. Since this species complex is the most confusing within the *Colletotrichum* genus, morphological identification alone cannot be trusted to identify the species correctly. It follows that the use of a polyphasic approach in plant pathology is crucial for the precise identification and naming of fungi, which will advance the management and control of both recognized and newly emerging diseases. [43]. Furthermore, a polyphasic approach using molecular analysis is an effective tool to identify cryptic species and estimate species diversity [49]. 

Among *Colletotrichum* species in the gloeosporioides species complex, *C. fructicola* has a very broad host range, isolated from more than eight plant families as endophytes and plant pathogens [48]. It has been reported from America, Asia, Africa, Europe, and Oceania [38]. In this study, we isolated and illustrated *C. fructicola* as the first report of *Colletotrichum* species associated with pineapple in Thailand, based on molecular and morphological analyses. Furthermore, the pathogenicity tests proved that the isolates are pathogens to pineapple (Figure 4).

The results of the present study can be useful for pathologists in understanding the fungal pathogen diversity associated with pineapple, disease management and quarantine purposes. Many new *Colletotrichum* species have been introduced in 2022 (https://www.mycobank.org/; accessed on 8 December 2022), and there are potentially many novel species of *Colletotrichum* that remained undiscovered [49]. Additionally, many studies reported new hosts for existing *Colletotrichum* species [50,51,52,53,54,55,56,57,58], broadening their host and geographical ranges. Therefore, more investigations on the isolation and identification of *Colletotrichum* associated with pineapple can improve our knowledge about fungal diversity and host range and will potentially lead to the discovery of novel species of *Colletotrichum*.

## 4. Materials and Methods

### 4.1. Sample Collection, Examination and Isolation

In order to isolate the fungal pathogens associated with pineapple plants, pineapple leaves with diebacks and leaf spots, and rotting fruits were considered. In total, 10 symptomatic leaves and 12 rotting pineapple fruits were collected during June–July 2022 from organic farms in Ban Du and Nang Lae sub-districts, Mueang Chiang Rai district, Chiang Rai province, northern Thailand. The samples were kept in plastic bags labelled with the collection date, collection site and host name before being transported to the laboratory for further examination. The fruiting bodies on natural substrates were observed and photographed using a stereomicroscope (OLYMPUS SZX16; Tokyo, Japan). Morphological features were observed using a LEICA-EZ4 stereomicroscope and photographed with an optical microscope equipped with a Nikon DS-Ri2 camera. The photo plates were made by the Adobe Photoshop v.21.1.2 software, and the scales were measured by the Tarosoft (R) Image Frame Work software.

Direct isolation and indirect isolation (tissue isolation) were used to obtain cultures [59]. Further, 30 mm^2^ leaf fragments were cut from the margins of lesions for tissue isolation, and were sterilized by submerging in 70% ethanol for 2 min, 10% sodium hypochlorite solution for 60 s, followed by three times rinsing in sterile distilled water for 60 s [32]. Following the procedures outlined by Senanayake et al., (2020), single-spore isolation and hyphal tip isolation were done to purify the isolates. Finally, the pure cultures were deposited in the Mae Fah Luang University Culture Collection (MFLUCC), Chiang Rai, Thailand. Specimens were deposited in the herbarium of the Center of Excellence in Fungal Research (CEFR), Mae Fah Luang University (MFLU).

### 4.2. DNA Extraction and PCR Amplification

Using a DNA Extraction Kit (Omega Biotek) in accordance with the manufacturer’s instructions, genomic DNA was extracted from fresh mycelia cultured on potato dextrose agar (PDA) for 14 days. The internal transcribed spacer (ITS), actin (*ACT*), chitin synthase (*CHS-1*), glyceraldehyde-3-phosphate dehydrogenase (*GAPDH*), and β-tubulin (*TUB2*) were amplified using primers ITS5/ITS4, ACT-512F/ACT-783R, CHS-79F/CHS-345R, GDF/GDR, and BT-2Fd/BT-4Rd, respectively (Table 2). The polymerase chain reaction was carried out in a total volume of 25 µL, including 12.5 µL of 2 × Power Taq PCR Master Mix, 1 µL of each primer (20 µM), 1 µL genomic DNA, and 9.5 µL of deionized water. The PCR procedure was done under the following conditions: Initial denaturation at 95 °C for 5 min, followed by 40 cycles of denaturation for 30 s at 95 °C; annealing at 53 °C for 60 s (ITS), 55 °C for 50 s (*ACT*), 58 °C for 30 s (*CHS-1*); 58 °C for 50 s (*GAPDH*), 58 °C for 90 s (*TUB2*); extension at 72 °C for 60 s; and the final extension at 72 °C for 10 min. PCR amplification was performed in an eppendorf thermal cycler (Master Cycler X50s). PCR products were sequenced by the SolGent company, Republic of Korea.

### 4.3. Phylogenetic Analyses

Sequences for the selected strains were obtained from GenBank (Table 3), according to blast-searching and related publications [40,41,42]. Multiple sequence alignments for ITS, *ACT*, *CHS-1*, *GAPDH*, and *TUB2* were constructed using MAFFT v.7.11 on the web server (https://mafft.cbrc.jp/alignment/server, accessed on 12 February 2023) with the default settings [64]. BioEdit v.7.0.9.0 was used for adjusting the sequences [65] and TrimAl software was used to trim aligned sequences automatically using the gappyout command. Maximum parsimony (MP) analysis was done using PAUP XSEDE [66]. Maximum likelihood (ML) analysis was performed on XSEDE with the GTR + Gamma model and 1000 replications using RAxML-HPC2 The Bayesian posterior probabilities analysis (BYPP) was carried out using a Markov Chain Monte Carlo (MCMC) algorithm using MrBayes on XSEDE [67]. In order to choose the best-fit evolutionary models for each dataset, jModeltest 2.1.10 and the Akaike Information Criterion (AIC) were employed on the CIPRES platform. Four MCMC chains were run from random trees for 1,000,000 generations and sampled every 100th generation. The first 25% of the generated trees were ignored as burn-in and the remaining trees were used for analyzing the posterior probabilities. Gaps were considered missing data, and ambiguously aligned parts were eliminated. The phylogenetic trees were visualized in FigTree v.1.4.0 [68], and were further edited in Adobe Illustrator CC 22.0.0 (Adobe Systems, San Jose, CA, USA).

### 4.4. Pathogenicity Assay

Koch’s postulates were applied according to the procedures demonstrated by Bhunjun et al., (2021) to confirm the pathogenicity of our isolates [69]. Three replicates of detached leaves from an organic farm were considered for both wounded and non-wounded assays using mycelial plug incubation because of the lack of culture sporulation. The pineapple leaves were surface sterilized by washing them in 70% ethanol for 2 min, then in 2% sodium hypochlorite for 2 min, followed by three washes with sterile distilled water and laminar air drying. We chose a pineapple leaf instead of fruit to accurately assess symptoms and fungal spread through the host. Mycelial plugs were obtained from the fresh colonies grown on PDA (10-day colonies). Control inoculations were performed using uninoculated PDA plugs. In a moist chamber at 28 °C and 80% relative humidity, the inoculated and control leaves were incubated. Koch’s postulates were confirmed by re-isolating the fungus from the infected leaves. The re-isolated fungus was identified based on cultural and morphological features.

## Figures and Tables

**Table 1:** Base pair differences between *C. fructicola* (ICMP 18581) and two newly isolated strains.

Isolate	ITS	*ACT*	*CHS*-*1*	*GAPDH*	*TUB2*
P76	2/511 bp	0/256 bp	0/241 bp	0/248 bp	1/402 bp
P76-3	3/511 bp	1/256 bp	0/241 bp	0/248 bp	0/402 bp

**Table 2:** Primers used in this study.

Gene	Primer	Sequence (5′ → 3′)	References
ITS	ITS 5 ITS 4	GGA AGT AAA AGT CGT AAC AAG G TCC TCC GCT TAT TGA TAT GC	[60]
*ACT*	ACT-512F ACT-783R	ATG TGC AAG GCC GGT TTC GC TAC GAG TCC TTC TGG CCC AT	[61]
*CHS-1*	CHS-79F CHS-345R	TGG GGC AAG GAT GCT TGG AAG AAG TGG AAG AAC CAT CTG TGA GAG TTG	[61]
*GAPDH*	GDF GDR	GCC GTC AAC GAC CCC TTC ATT GA GGG TGG AGT CGT ACT TGA GCA TGT	[62]
*TUB2*	BT-2Fd BT-4Rd	GTB CAC CTY CAR ACC GGY CAR TG CCR GAY TGR CCR AAR ACR AAG TTG TC	[63]

**Table 3:** Taxa and their GenBank accession numbers used in the phylogenetic analysis.

Taxa	Strains			GenBank Accession Numbers		
		ITS	*GAPDH*	*CHS-1*	*ACT*	*TUB*
*Colletotrichum acidae*	MFLUCC 17-2659 *	MG996505	MH003691	MH003694	MH003697	MH003700
*C. aenigma*	ICMP 18608 *	JX010244	JX010044	JX009774	JX009443	JX010389
*C. aeschynomenes*	ICMP 17673 *, ATCC 201874	JX010176	JX009930	JX009799	JX009483	JX010392
*C. alatae*	CBS 304.67 *, ICMP 17919	JX010190	JX009990	JX009837	JX009471	JX010383
*C. alienum*	ICMP 12071 *	JX010251	JX010028	JX009882	JX009572	JX010411
*C. aotearoa*	ICMP 18537 *	JX010205	JX010005	JX009853	—	JX010420
*C. arecicola*	CGMCC 3.19667 *	MK914635	—	MK935541	MK935374	MK935498
*C. artocarpicola*	MFLUCC 18-1167 *	MN415991	MN435568	MN435569	MN435570	MN435567
*C. asianum*	ICMP 18580 *, CBS 130418	JX010196	JX010053	JX009867	JX009584	JX010406
*C. analogum*	YMF1.06943 *	OK030860	OK513663	OK513559	OK513599	OK513629
*C. camelliae*	CGMCC 3.14925, LC1364 *	KJ955081	KJ954782	MZ799255	KJ954363	KJ955230
*C. cangyuanensis*	YMF1.05001 *	OK030864	OK513667	OK513563	OK513603	OK513633
*C. changpingense*	CGMCC 3.17582 *, SA0016, MFLUCC 15-0022	KP683152	KP852469	KP852449	KP683093	KP852490
*C. chiangmaiense*	MFLUCC 18-0945 *	MW346499	MW548592	MW623653	MW655578	—
*C. chrysophilum*	URM 7368, CMM4268 *	KX094252	KX094183	KX094083	KX093982	KX094285
*C. cigarro*	ICMP 18539 *	JX010230	JX009966	JX009800	JX009523	JX010434
*C. clidemiae*	ICMP 18658 *	JX010265	JX009989	JX009877	JX009537	JX010438
*C. cobbittiense*	BRIP 66219 *	MH087016	MH094133	MH094135	MH094134	MH094137
*C. conoides*	CGMCC 3.17615, CAUG17, LC6226 *	KP890168	KP890162	KP890156	KP890144	KP890174
*C. cordylinicola*	MFLUCC 090551 *, ICMP 18579	JX010226	JX009975	JX009864	HM470234	JX010440
*C. dimorphum*	YMF1.07309 *	OK030867	OK513670	OK513566	OK513606	OK513636
*C. dracaenigenum*	MFLUCC 19-0430 *	MN921250	MT215577	MT215575	MT313686	—
*C. endophytica*	MFLUCC 13-0418, LC0324 *	KC633854	KC832854	MZ799261	KF306258	MZ673954
*C. fici-septicae*	MFLU 19-2770 *	MW114367	MW183774	MW177701	MW151585	—
*C. fructicola*	ICMP 18581 *, CBS 130416	JX010165	JX010033	JX009866	FJ907426	JX010405
*C. fructicola*	MFLUCC 17-0555	MG646969	MG646936	MG646932	MG646944	MG646928
*C. fructicola*	MFLUCC 17-0613	MG646968	MG646935	MG646933	MG646939	MG646927
***C. fructicola***	**P76,** **MFLUCC 22-0181**	**OQ048649**	**OQ067350**	**OQ067349**	**OQ067348**	**OQ067351**
***C. fructicola***	**P76-3,** **MFLUCC 22-0182**	**OQ048650**	**OQ067354**	**OQ067353**	**OQ067352**	**OQ067355**
*C. fructivorum*	Coll1414, BPI 884103, CBS133125 *	JX145145	MZ664047	MZ799259	MZ664126	JX145196
*C. gloeosporioides*	IMI 356878 *, ICMP 17821, CBS 112999	JQ005152	JQ005239	JQ005326	JQ005500	JQ005587
*C. gracile*	YMF1.06939 *	OK030868	OK513671	OK513567	OK513607	OK513637
*C. grevilleae*	CBS 132879, CPC 15481 *	KC297078	KC297010	KC296987	KC296941	KC297102
*C. grossum*	CGMCC 3.17614, CAUG7, LC6227 *	KP890165	KP890159	KP890153	KP890141	KP890171
*C. hebeiense*	MFLUCC 13-0726 *	KF156863	KF377495	KF289008	KF377532	KF288975
*C. hederiicola*	MFLU 15-0689 *	MN631384	—	MN635794	MN635795	—
*C. helleniense*	CBS 142418, CPC 26844 *	KY856446	KY856270	KY856186	KY856019	KY856528
*C. henanense*	LC3030, CGMCC 3.17354, LF238 *	KJ955109	KJ954810	MZ799256	KM023257	KJ955257
*C. horii*	NBRC 7478 *, ICMP 10492, MTCC 10841	GQ329690	GQ329681	JX009752	JX009438	JX010450
*C. hystricis*	CBS 142411, CPC 28153 *	KY856450	KY856274	KY856190	KY856023	KY856532
*C. jiangxiense*	CGMCC 3.17363 *	KJ955201	KJ954902	—	KJ954471	KJ955348
*C. kahawae*	IMI 319418 *, ICMP 17816	JX010231	JX010012	JX009813	JX009452	JX010444
*C. makassarense*	CBS 143664 *	MH728812	MH728820	MH805850	MH781480	MH846563
*C. musae*	CBS 116870 *, ICMP 19119, MTCC 11349	HQ596292	HQ596299	JX009896	HQ596284	HQ596280
*C. nanhuaensis*	YMF1.04993 *	OK030870	OK513673	OK513569	OK513609	OK513639
*C. nullisetosum*	YMF1.06946 *	OK030872	OK513675	OK513571	OK513611	OK513641
*C. nupharicola*	CBS 470.96 *, ICMP 18187	JX010187	JX009972	JX009835	JX009437	JX010398
*C. oblongisporum*	YMF1.06938 *	OK030874	OK513677	OK513573	—	OK513643
*C. parvisporum*	YMF1.06942 *	OK030876	OK513679	OK513575	OK513613	OK513645
*C. pandanicola*	MFLUCC 17-0571 *	MG646967	MG646934	MG646931	MG646938	MG646926
*C*. *perseae*	CBS 141365 *, GA100	KX620308	KX620242	MZ799260	KX620145	KX620341
*C. proteae*	CBS 132882 *	KC297079	KC297009	KC296986	KC296940	KC297101
*C. pseudotheobromicola*	MFLUCC 18-1602 *	MH817395	MH853675	MH853678	MH853681	MH853684
*C. psidii*	CBS 145.29 *, ICMP 19120	JX010219	JX009967	JX009901	JX009515	JX010443
*C. queenslandicum*	ICMP 1778 *	JX010276	JX009934	JX009899	JX009447	JX010414
*C. rhexiae*	Coll1026, BPI 884112, CBS 133134 *	JX145128	MZ664046	MZ799258	MZ664127	JX145179
*C. salsolae*	ICMP 19051 *	JX010242	JX009916	JX009863	JX009562	JX010403
*C. siamense*	ICMP 18578 *, CBS 130417	FJ972613	FJ972575	JX009865	FJ907423	FJ907438
*C. syzygiicola*	DNCL021, MFLUCC 10-0624 *	KF242094	KF242156	—	KF157801	KF254880
*C. tainanense*	CBS 143666 *	MH728818	MH728823	MH805845	MH781475	MH846558
*C. temperatum*	CBS 133122 *, Coll883, BPI 884100	JX145159	MZ664045	MZ799254	MZ664125	JX145211
*C. tengchongense*	YMF 1.04950 *	OL842169	OL981264	OL981290	OL981238	—
*C. theobromicola*	CBS 124945 *, ICMP 18649	JX010294	JX010006	JX009869	JX009444	JX010447
*C. ti*	ICMP 4832 *	JX010269	JX009952	JX009898	JX009520	JX010442
*C. tropicale*	CBS 124949 *, ICMP 18653, MTCC 11371	JX010264	JX010007	JX009870	JX009489	JX010407
*C. truncatum*	CBS 151.35 *	GU227862	GU228254	GU228352	GU227960	GU228156
*C. viniferum*	GZAAS 5.08601 *, yg1	JN412804	JN412798	—	JN412795	JN412813
*C. vulgaris*	YMF 1.04940 *	OL842170	OL981265	OL981291	OL981239	—
*C. wuxiense*	CGMCC 3.17894 *	KU251591	KU252045	KU251939	KU251672	KU252200
*C. xanthorrhoeae*	BRIP 45094 *, ICMP 17903, CBS 127831	JX010261	JX009927	JX009823	JX009478	JX010448
*C. xishuangbannaense*	MFLUCC.19-0107 *	MW346469	MW537586	MW660832	MW652294	—
*C. yulongense*	CFCC.50818 *	MH751507	MK108986	MH793605	MH777394	MK108987
*C. yunajiangensis*	YMF1.04996 *	OK030885	OK513686	OK513583	OK513620	OK513649

Type strains are indicated with “*”. The isolates sequenced in this study are in bold.

## Data Availability

Publicly available datasets were analyzed in this study. These data can be found here: https://www.ncbi.nlm.nih.gov (accessed on 12 November 2022).
